# Assessment of a wireless headband for automatic sleep scoring

**DOI:** 10.1007/s11325-012-0757-4

**Published:** 2012-09-21

**Authors:** H. Griessenberger, D. P. J. Heib, A. B. Kunz, K. Hoedlmoser, M. Schabus

**Affiliations:** 1 Department of Psychology, Laboratory for Sleep, Cognition and Consciousness Research, University of Salzburg, Hellbrunnerstrasse 34, 5020 Salzburg, Austria; 2 Christian Doppler Clinic, Department of Neurology, Paracelsus Private Medical University, Ignaz-Harrerstraße 79, 5020 Salzburg, Austria; 3 Laboratory for Sleep, Cognition and Consciousness Research, Department of Psychology, University of Salzburg, Hellbrunnerstrasse 34, 5020 Salzburg, Austria

**Keywords:** Sleep staging, Wireless headband, EEG, Sleep disturbance

## Abstract

**Purpose:**

Classically, professional assessment of sleep is done in the sleep laboratory using whole-night polysomnography (PSG). However, given a misbalance between accredited sleep laboratories and the large amount of patients suffering from sleep disorders, only few receive appropriate diagnostic assessment. Recently, some low-cost home sleep scoring systems have been proposed, yet such systems are rarely tested scientifically. The aim of the present study was to evaluate the staging accuracy of the home sleep scoring system Zeo (Newton, MA, USA).

**Methods:**

A final sample of 21 nights from ten subjects (aged 23–45) was digitally recorded with PSG as well as with the Zeo system. We compared scorings of Zeo (on an epoch-be-epoch basis) with the Somnolyzer 24 × 7 (an automatic staging algorithm), expert scorers as well as the freeware SleepExplorer.

**Results:**

It was revealed that Zeo shows moderate overall agreement as compared to our study standard Somnolyzer 24 × 7 (*κ* = 0.56). The most obvious performance difference between Zeo and both other scoring approaches was stage wake (sleep onset latency + wake after sleep onset). While Zeo detected only 40.8 % of the study standard wake epochs, 70.1 % were detected by the expert scorers and 83.4 % by the SleepExplorer, respectively.

**Conclusions:**

Data suggest that the Zeo system produces acceptable sleep scoring for stage REM, light and deep sleep, with a specific weakness in correctly detecting waking periods.

**Electronic supplementary material:**

The online version of this article (doi:10.1007/s11325-012-0757-4) contains supplementary material, which is available to authorized users.

## Introduction

According to the most recent criteria of the American Academy of Sleep Medicine [1], at least three electroencephalogram (EEG) channels, two unipolar electrooculogram (EOG) and three electromyogram (EMG) channels are needed for appropriate measurement of sleep stages. This standard polysomnographic setup requires expensive equipment as well as trained personnel and can therefore only be performed in professional sleep laboratories. The main advantage of a laboratory-based sleep assessment is the on-site presence of health care professionals checking for various sleep disorders as well as the control for environmental variables. Sleeping in a laboratory setting however is unusual for subjects and can therefore result in altered sleep architecture, such as present in the well-known first night effect (e.g. [2, 3]). Furthermore, the high financial costs, the low availability of sleep laboratories and the long waiting periods strongly restrict the number of consecutive nights in the sleep laboratory. Unfortunately, many sleep disorders do not occur on a daily basis, and it is therefore likely that disorders are missed if only one to two nights can be assessed. Hence, other technologies have been developed and tested for home sleep applicability ([4–6], for review see [7]). These alternative devices are systems specialized for detection of certain sleep disorders (e.g. obstructive sleep apnea [8, 9]). On the other hand, a software for automatic sleep scoring (e.g. ASEEGA system [10]) or detection of sleep/wake states by radiofrequency biomotion sensors [11] provides reliable alternatives to classic polysomnography.

In the present study, a wireless home sleep monitoring system was evaluated for a mixed group of (subclinical) insomnia patients and good sleeper controls. The study aim was an objective external evaluation, which discusses weaknesses and strengths of the Zeo system. We leave it open to the reader to conclude the applicability to his or her field of interest. Specifically, we studied the Zeo system (Axon Laboratories; Newton, MA, USA) [12, 13] which scores the night in 30-s epochs and four stages (wake, light sleep, deep sleep and rapid eye movement sleep) simply using three dry frontal electrodes. Data are compared with our study standard Somnolyzer 24 × 7 [14, 15]. Additionally, we report the comparison scorings between our study standard and both an expert scorer (A.K.) and the automatic sleep analysis of the freeware SleepExplorer (El Ratón Networks). Therefore, we tested the Zeo system against an automatic (SleepExplorer), semi-automatic (Somnolyzer) and manual sleep staging.

## Methods

Data were obtained from a larger study cohort where people suffering from primary insomnia were compared to good sleeper controls (age range = 20–57). All participants were requested to be non-habitual smokers (less than five cigarettes a day). A preceding entrance examination included Diagnosis of Psychiatric Disorders according to DSM IV (Structured Clinical Interview for DSM Disorders) and psychometric tests (e.g. personality questionnaires). Patients (primary insomnia) were classified according to the research diagnostic criteria of Edinger and colleagues [16]. Healthy controls were regular sleepers which was determined by clinical interviews and sleep quality questionnaires (Pittsburgh Sleep Quality Index score <5). Originally, 37 records (nights) of 13 subjects were included in this study. Due to technical difficulties (reoccurring data loss over the night in the Zeo data recordings), only 21 recordings could be analyzed. The remaining records had to be excluded as synchronization between polysomnographic EEG data and Zeo data was not possible in an epoch-by-epoch manner. Finally, each recording consisted of a minimum of 6 h (720 epochs) of undisturbed sleep stages. Participants provided a written informed consent. The electroencephalogram (EEG) was recorded utilizing Synamps EEG amplifiers (NeuroScan Inc., El Paso, Texas). All signals were filtered (0.10 Hz high-pass filter; 70 Hz low-pass filter; 50 Hz notch filter) and digitized online with 500 Hz sampling rate. Twenty-three EEG channels (Fp1, Fpz, Fp2, F7, F3, Fz, F4, F8, T3, C3, Cz, C4, T4, T5, P3, Pz, P4, T6, O1, Oz, O2, as well as A1 and A2 for later re-referencing), four electrooculogram (EOG) channels, one bipolar submental electromyogram (EMG) channel, one bipolar electrocardiogram (ECG) channel and one bipolar respiratory channel (chest wall movements) were placed. Electrodes were attached according to the international electrode (10–20) placement system (cf. [17]).

All recordings were scored by four different systems: (1) the semiautomatic scoring of Somnolyzer 24 × 7 (serving as our study standard [14]), (2) the manual scoring of one expert scorer (A.K.) (according to AASM rules [1]), (3) the automatic pre-scoring of the freeware software SleepExplorer (El Ratón networks, http://www.sleepexplorer.de/) and (4) the Zeo device (instrument of interest). Expert scorers were blind to the outcome of the other utilized methods.

We decided to use the semi-automatic Somnolyzer 24 × 7 as our study standard due to the reported reliable sleep classification which is also manually reviewed and revised if needed. Moreover, it is to be noted that reliability scores were shown to be better between Somnolyzer 24 × 7 and manual scorers than the reliability between human scorers [14] (also see Supplementary Table 1).

The Zeo headband consisted of three frontal wireless dry electrodes on a lightweight headband which was placed at the forehead—below electrodes Fp1, Fpz, Fp2. Like defined by classical staging criteria, the Zeo system scores a night in 30-s epochs but only in four stages (wake, light sleep, deep sleep and rapid eye movement sleep). Yet note that the underlying ZEO scoring algorithms are unfortunately proprietary and not open for in-depth evaluation.

### Epoch-by-epoch comparison

For each recording, raw data were synchronized so that the onset of the first epoch was identical for all scoring systems. Epoch synchronization was also corrected for EEG data loss in case of bathroom visits or electrode adjustments during the night. The four different scoring systems were tested for their agreement on the following four stages: [wake (sleep onset latency (SOL) + wake after sleep onset (WASO)), light sleep (N1 or N2), deep sleep (N3) and rapid eye movement sleep (REM)].

## Statistical analyses

Statistical analyses were performed using PASW 18.0.0 software (SPSS Inc., Chicago, IL). The significance level was set to *p* < 0.05. The outcome of Somnolyzer 24 × 7 stagings (defined as study standard) was compared against the other three approaches with a focus on the Zeo system.

Epoch by epoch agreement was defined as the percentage of epochs that were assigned the same stage. Paired sample *t* tests were calculated for percentage agreements to the study standard for overall agreement, stages wake, light sleep, deep sleep and rapid-eye-movement sleep. We also calculated sensitivity and positive predictive values (PPV).

Cohen’s kappa (*κ*) was used to assess the agreement for pairwise comparisons. Partial correlation coefficients, controlling for the total number of epochs, were used for quantitative analysis. Bland–Altman plots served for visualizing agreement between two given scoring approaches. Comparisons were done for our study standard versus Zeo, expert scorer and SleepExplorer analysis.

## Results

The mean age of the final study sample was 32.5 years (SD = 7.63; range, 23–45). Three of the subjects reported no sleep disorder (in total six recordings), whereas seven subjects were suffering from primary insomnia.

According to our study standard, time spent in bed was 470.07 min (SD = 15.76 min), total sleep time was 435.14 min (SD = 29.48 min) and sleep efficiency was 92.5 % (SD = 4.48 %). On average, the subjects spent 85.98 min (SD = 30.83 min) in deep sleep (N3), 256.74 min (SD = 45.76 min) in light sleep (N1+N2) and 92.43 min (SD = 27.09 min) in REM sleep. SOL was 10.79 min (SD = 11.18 min), and WASO was 24.14 min (SD = 12.1 min). Table 1 illustrates the percent agreement of the Zeo system with all other methods across the 21 recorded nights (for a contingency table based on each epoch, see Supplemental Table 1).

**Table 1:** Percent agreement table for epoch labeling between Zeo and comparison scorings (Somnolyzer 24 × 7, Expert and SleepExplorer)

		Zeo			
		Wake (%)	REM (%)	Light (%)	Deep (%)
Somnolyzer 24 × 7	Wake	40.83	26.79	29.24	3.14
	REM	11.54	73.69	13.40	1.37
	Light	7.21	9.17	80.04	3.59
	Deep	1.30	0.08	36.48	62.13
Expert scorer	Wake	40.05	24.03	30.96	4.96
	REM	13.94	70.65	14.28	1.13
	Light	4.88	9.46	79.13	6.52
	Deep	0.48	0.67	29.94	68.91
SleepExplorer	Wake	31.39	30.93	34.01	3.67
	REM	13.39	67.77	18.00	0.83
	Light	2.89	4.62	84.93	7.56
	Deep	0.86	0.49	29.45	69.20

### Stage comparisons

The overall agreement (all 19,738 epochs from all subjects) was 72.6 % (*κ* = 0.56) between Somnolyzer 24 × 7 and Zeo, 80.9 % (*κ* = 0.69) between Somnolyzer 24 × 7 and expert scorer and 74.2 % (*κ* = 0.61) between Somnolyzer 24 × 7 and SleepExplorer. Paired sample *t* tests revealed a significant difference between the Zeo and expert scorer agreements (*t* = −4.048; *p* < .01) indicating a significant higher agreement score for the expert scorer with the study standard. Similar differences were revealed between the expert and SleepExplorer agreements (*t* = 5.204; *p* < .01) indicating better overall agreements with the study standard for the expert scorer.

All PPV and scores for sensitivity are reported in Table 2. Zeo revealed the worst sensitivity values in stage wake (40.8 %) indicating that less than half of the study standard wake epochs are correctly detected. Best scoring agreement was represented by expert scorer in light sleep (86.4 %).

**Table 2:** Classification results between Somnolyzer 24 × 7 and comparison scorings (Zeo, Expert and SleepExplorer)

			Wake	REM	Light	Deep
Zeo						
% OA	72.6	Sensitivity	40.8	73.7	80.0	62.1
Cohen’s kappa	0.56	PPV	32.0	67.4	79.2	82.2
Expert						
% OA	80.9	Sensitivity	70.1	80.1	86.4	69.8
Cohen’s kappa	0.69	PPV	53.1	81.6	82.4	93.7
SleepExplorer						
% OA	74.2	Sensitivity	83.4	74.2	75.3	67.2
Cohen’s kappa	0.61	PPV	37.7	70.2	83.4	91.3

*% OA* percent overall agreement, *sensitivity* percentage of actual sleep state epochs that are labelled as sleep state epochs by classifier, *PPV* positive predictive value (percentage of epochs labelled as sleep state that are correctly labelled)

### Bland and Altman plots

**Fig. 1:**
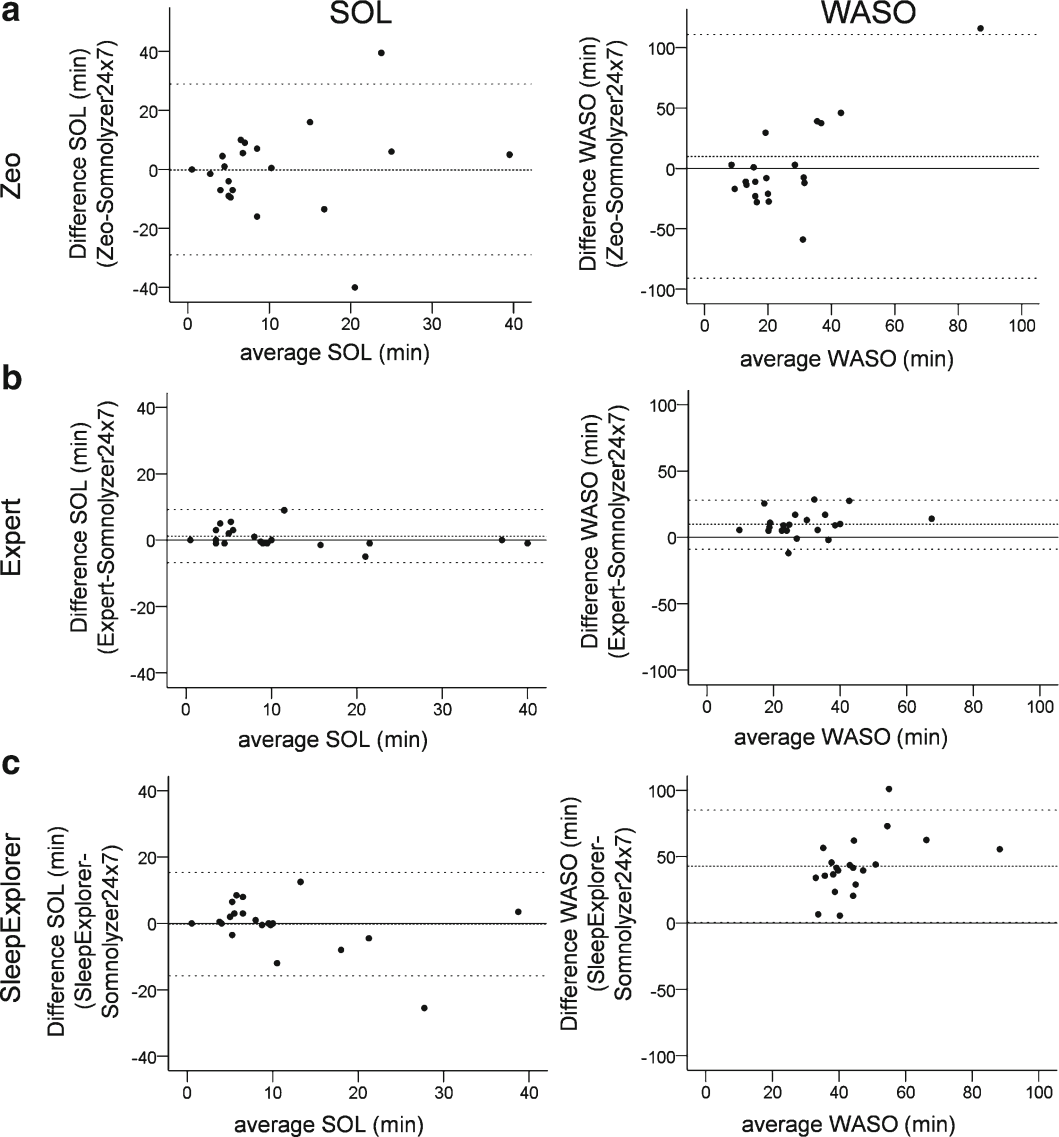
Bland–Altman plots of sleep parameters SOL and WASO showing differences between Somnolyzer 24 × 7 and **a** Zeo, **b** expert **c** SleepExplorer scorings. The *x*-axes indicate the average from both the study standard and comparison scoring of wake after sleep onset and sleep onset latency. The difference is expressed as the comparison score minus the study standard score. The mean difference and the limits of agreement (±1.96 SD) are represented as *dashed lines*. Note that high variabilities are dominant for Zeo scorings

Figure 1 shows the Bland–Altman plots visualizing the agreements of Somnolyzer 24 × 7 and the three other scoring instruments for wake parameters (SOL + WASO). There are no systematic under- or overestimation of the Zeo scorings (see Supplemental material). The greatest mean underestimation (21 min) of Zeo as compared to the study standard is revealed for deep sleep, which reaches significance (*t*_20_ = 4.225; *p* < 0.001). As shown in Fig. 1a, high variability for waking stages, that is in SOL and WASO, is evident. Yet, note that epoch-by-epoch agreement is better reflected in Table 1.

### Correlations

**Fig. 2:**
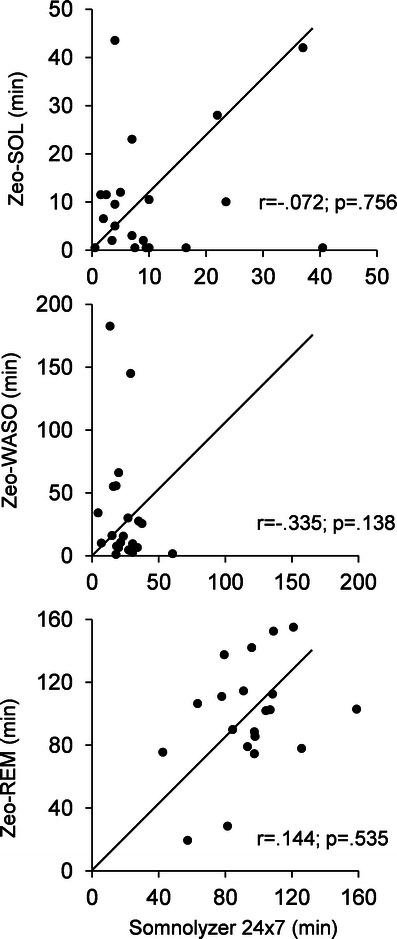
Scatter plots depicting the agreement of Somnolyzer 24 × 7 with Zeo for SOL, WASO and REM (*top to bottom*). Note that the SOL agreement for Zeo and study standard shows high deviations. *Dots* are scattered around the 45° line of identity

Correlations between Somnolyzer 24 × 7 and Zeo stage minutes revealed significant results for light sleep (*r* = .480, *p* < 0.05) and deep sleep (*r* = .695, *p* < 0.01). SOL, WASO and REM sleep were not significantly related. For a better overview, Fig. 2 depicts this mismatch. Significant correlations between expert scorer and Somnolyzer 24 × 7 were found in light sleep (*r* = .680, *p* < 0.01), deep sleep (*r* = .856, *p* < 0.01), SOL (*r* = .951, *p* < 0.01) and WASO (*r* = .634, *p* < 0.01). The automatic sleep analysis software SleepExplorer showed significant study standard correlations in SOL (*r* = .714, *p* < 0.01), light sleep (*r* = .561, *p* < 0.05), deep sleep (*r* = .693, *p* < 0.01) and REM sleep (*r* = .633, *p* < 0.05).

## Discussion

The results of this evaluation study show that the Zeo system provides a moderate sleep scoring with an overall agreement of 72.7 %. A Kappa coefficient of 0.56 indicates a reasonable agreement according to Landis and Koch [18]. Zeo revealed the worst sensitivity in stage wake (40.8 % epoch-by-epoch agreement; cf. Table 1), a mean underestimation of deep sleep of 21 min and additionally low correlation coefficients for SOL and WASO (cf. Fig. 1). Best agreement was revealed for light sleep (80 %).

In comparison to Zeo, the overall agreement of the expert scorer was 80.9 %, and interestingly, even the freely available automatic analysis tool SleepExplorer revealed an agreement of 74.2 %. The worst sensitivity for expert scorer and sleep explorer tool was stage deep sleep (69.81 %; 67.23 %). It has to be fairly taken into account that the latter two scorings rely on full PSGs, whereas the Zeo system has to derive its values from three dry, prefrontal electrodes.

To put the results in context, the wireless sleep assessment system Zeo appears to perform similar to other home sleep monitoring systems. Existing actigraphy systems provide epoch-by-epoch accuracies for sleep–wake separation of 75–85 % [19]. Devices which use just a single EEG channel show an accuracy of 84.9 % (i.e. [10]). Earlier reported in-house Zeo investigation revealed epoch-per-epoch accuracies between 73 and 91 % [12, 13]. Moreover, the agreement of stage wake was 64 % in these studies as compared to our findings of 40.8 %. This difference might also be due to our more inhomogeneous testing sample (healthy as well as primary insomnia patients). On the other hand, sleep architecture proved to be indistinguishable between the two groups, which is also related to the fact that primary insomnia is often found to be a subjective rather than objective complaint [20].

Although the Zeo results can be viewed as acceptable given the ultra quick and easy handling of the device, it is necessary to consider that only about half of all recorded nights could be used for statistical analyses. The rest of the data had too many missing values, mainly because of losing the headband during the night. On the other hand, one can certainly argue that there is no reason for not recording a week or more of data with the Zeo until a reliable set of data has been acquired. Specifically, it is evident that the Zeo system shows weak detection rates for parameters SOL and WASO and as a consequence of stage wake. As these values are often key features when assessing sleep quality, it is necessary to be specifically cautious in interpreting these Zeo measures. Due to the fact that Zeo does not systematically under- or overestimate waking, it is difficult to interpret provided SOL and WASO values.

A potential issue of our study is the unequal number of analyzed nights from patients and healthy volunteers. Yet, we believe that just a mixed real-world sample of that kind is ideal for testing sleep scoring systems: what is still missing is a full-blown evaluation study of home-based systems such as the Zeo together with ambulatory PSG. One big advantage of home-based systems might be the assessment of more habitual sleep at home, especially in groups of subjects which are hard to study otherwise (elderly and children).

One other limitation of this study may be the fact that at present we only tested PSG nights of healthy sleepers as well as primary insomnia patients. It is important to check if results would differ more if patients with sleep disorders other than insomnia are included (e.g. periodic limb movement disorders, restless legs syndrome, parasomnia and sleep apnea). The present study cannot directly address these questions and has to leave this evaluation open to future studies. Just for the integration of multiple channel recordings (EOG, EMG and EEG) expert knowledge seems to be most solid. We also expect that automatic classifiers might have serious problems when it comes to bad PSG data quality, whereas expert scorers might still be able to extract the critical sleep features to assess sleep reliably. Yet this point has to be addressed in the future and is open to discussion.

In summary, moderate sensitivity and positive predictive value scores were revealed for the home-based Zeo system with stage waking showing the biggest deviance from our study standard. Therefore, systems like Zeo might have a promising future if they can overcome limitations such as data loss during the night or insufficient SOL and WASO detection. Yet, due to the weak detection rates for SOL and WASO, systems of that kind cannot be suggested for home-based diagnostic of sleep disorders to date. Further improvements are needed in order to allow that practical devices such as the Zeo system can be recommended complimentary to sleep diaries and even to assist the clinical decision process for a variety of sleep disorders. Objective evaluation and independent studies of various groups are however inevitable if these systems intend to be approved by scientific standards.

People simply interested in getting a rough picture of their sleep over weeks might already benefit today by utilizing inexpensive sleep scoring devices such as the Zeo system. Manufacturers, however, should communicate limitations more readily and provide information regarding possible shortcomings such as insufficient wake detection or sleep onset latency.

Any tool to enhance awareness of sleep hygiene would greatly benefit our health in a society in which workplace demands and chronic stress induce unhealthy sleep habits. Today such reliable home-based sleep scoring systems are still awaited.

## Electronic supplementary material

Below is the link to the electronic supplementary material.

ESM 1 (JPEG 939 kb)

High resolution image (TIFF 2229 kb)

Supplementary Table 1 (JPEG 983 kb)

High resolution image (TIFF 1358 kb)

Supplementary Table 2 (JPEG 1658 kb)

High resolution image (TIFF 2280 kb)
