# Supplementary figures and images for: Assessment of a wireless headband for automatic sleep scoring
Source: Sleep Breath. 2012 Sep 21;17(2):747–52. doi: 10.1007/s11325-012-0757-4 (PMC3655221; doi:10.1007/s11325-012-0757-4)

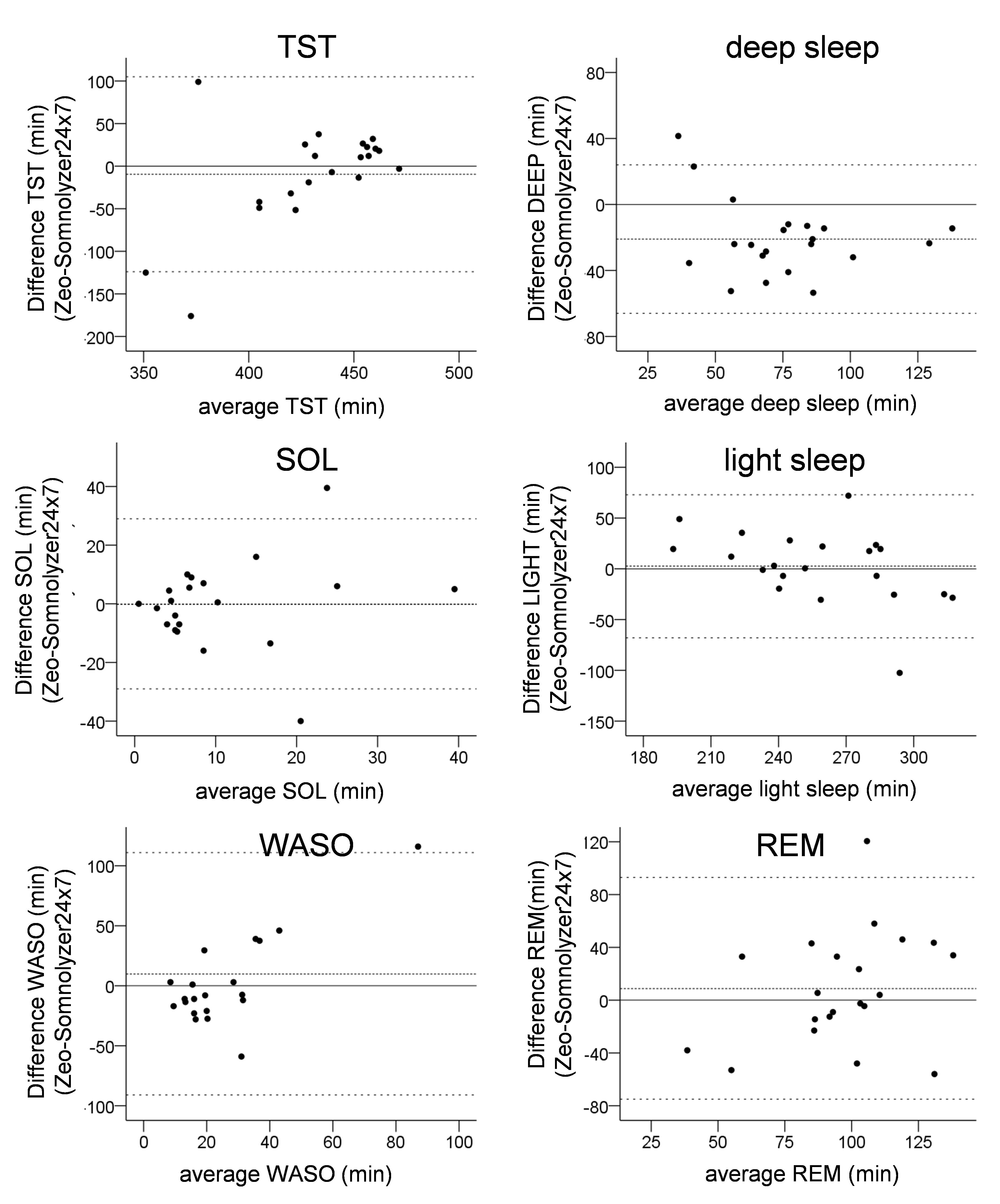

Supplement: Supplementary file 1 — (JPEG 939 kb) [file 11325_2012_757_Fig3_ESM.jpg]

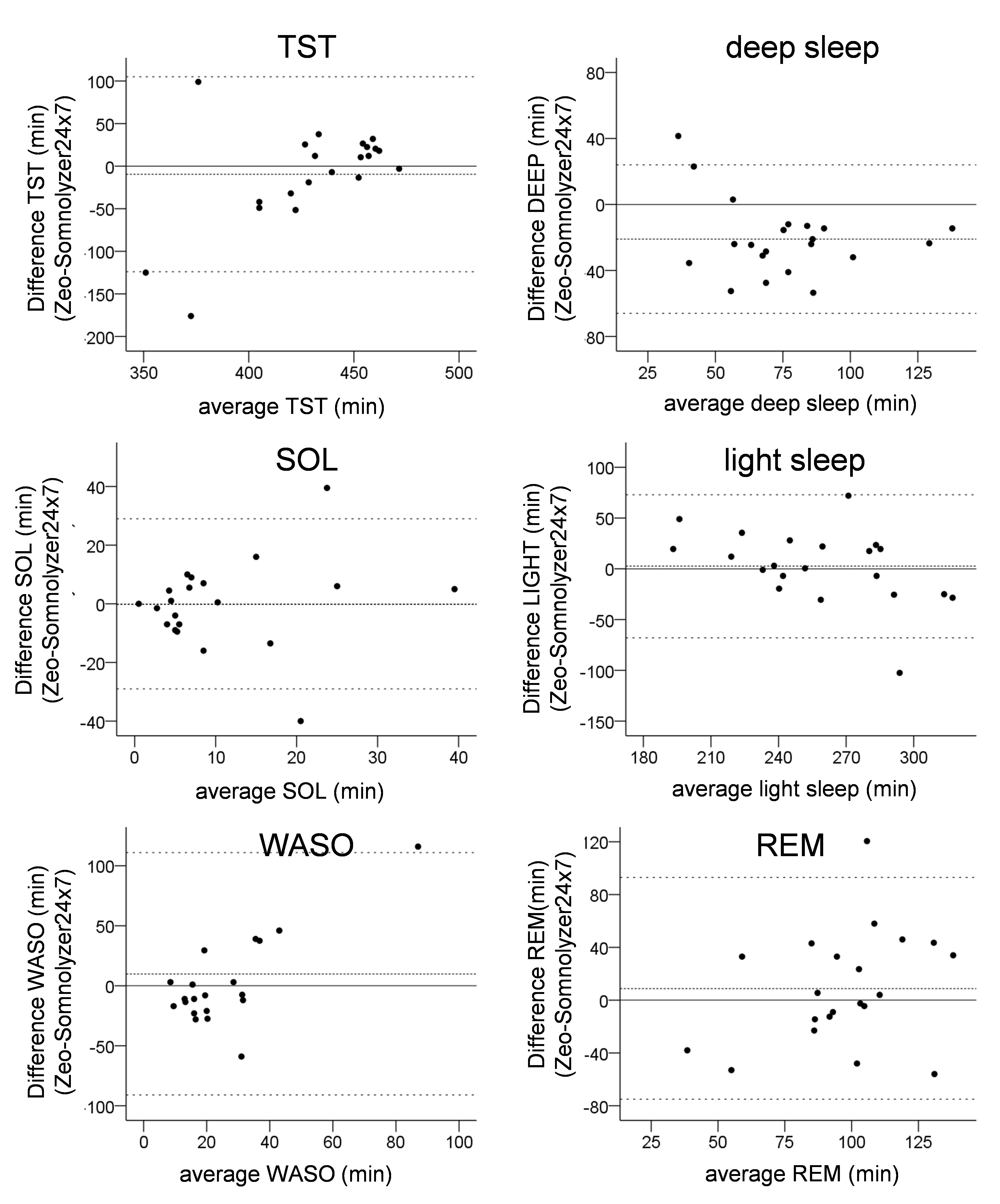

Supplement: Supplementary file 2 — High resolution image (TIFF 2229 kb) [file 11325_2012_757_MOESM1_ESM.tif]

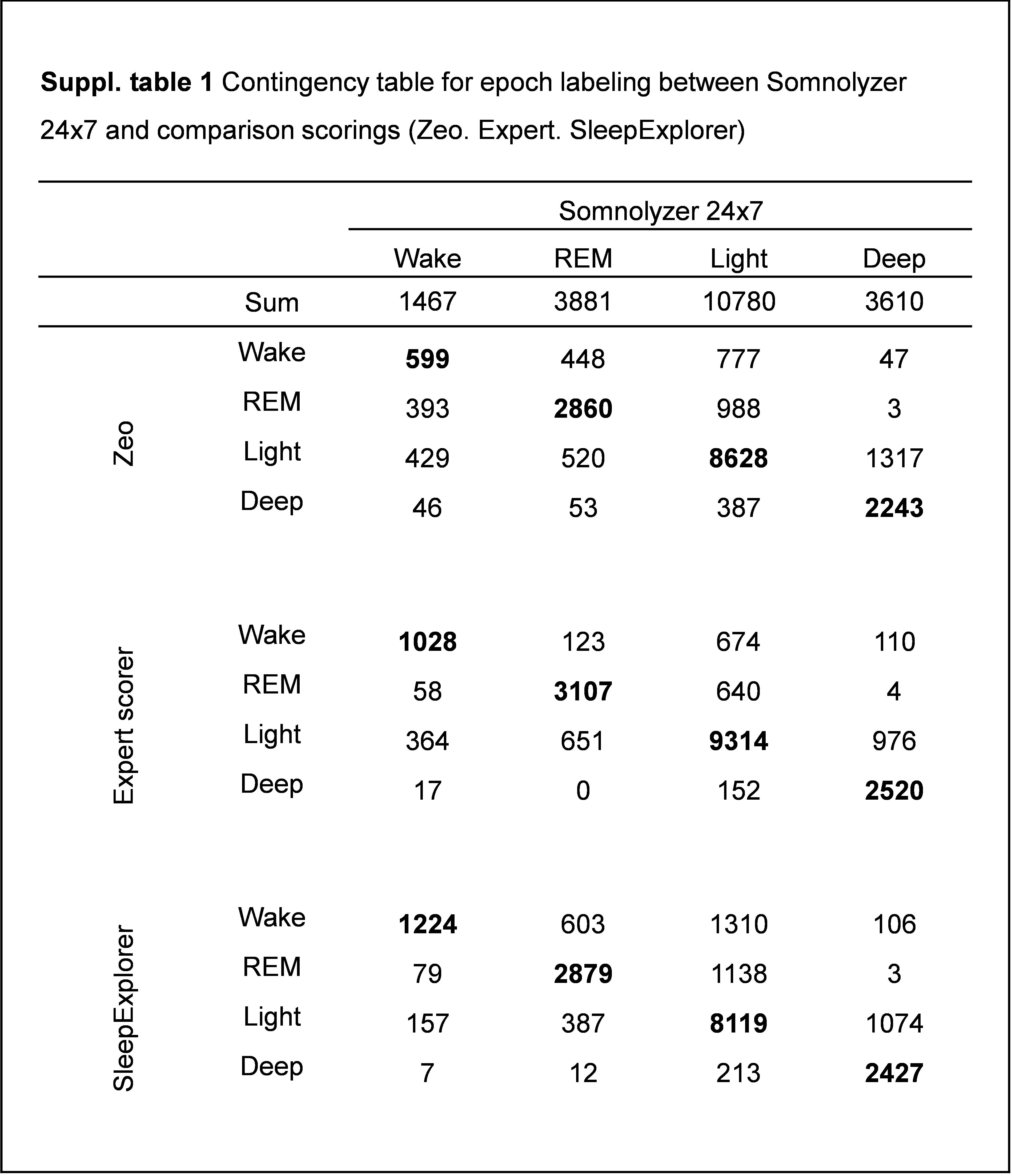

Supplement: Supplementary file 3 — (JPEG 983 kb) [file 11325_2012_757_Fig4_ESM.jpg]

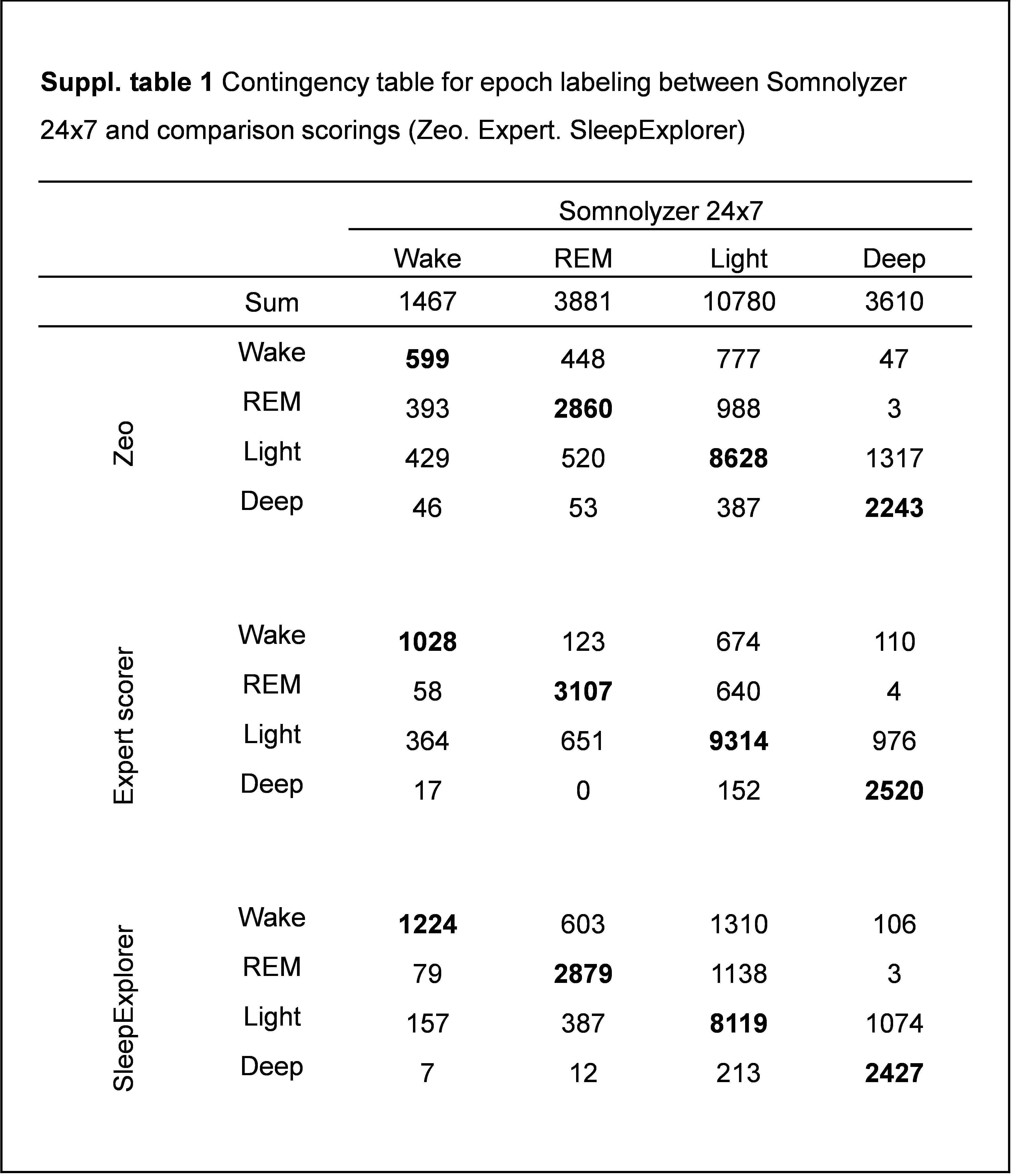

Supplement: Supplementary file 4 — High resolution image (TIFF 1358 kb) [file 11325_2012_757_MOESM2_ESM.tif]

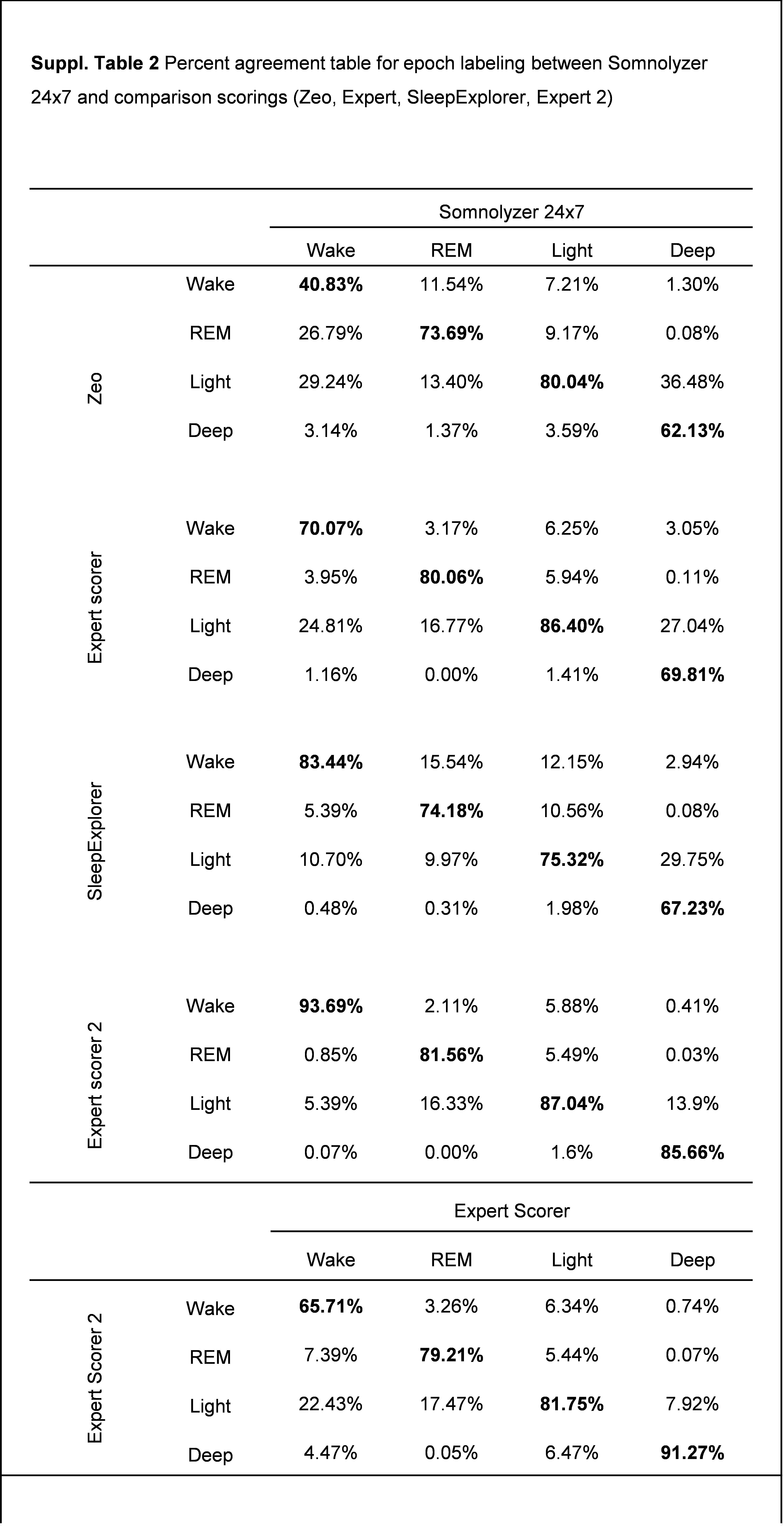

Supplement: Supplementary file 5 — (JPEG 1658 kb) [file 11325_2012_757_Fig5_ESM.jpg]

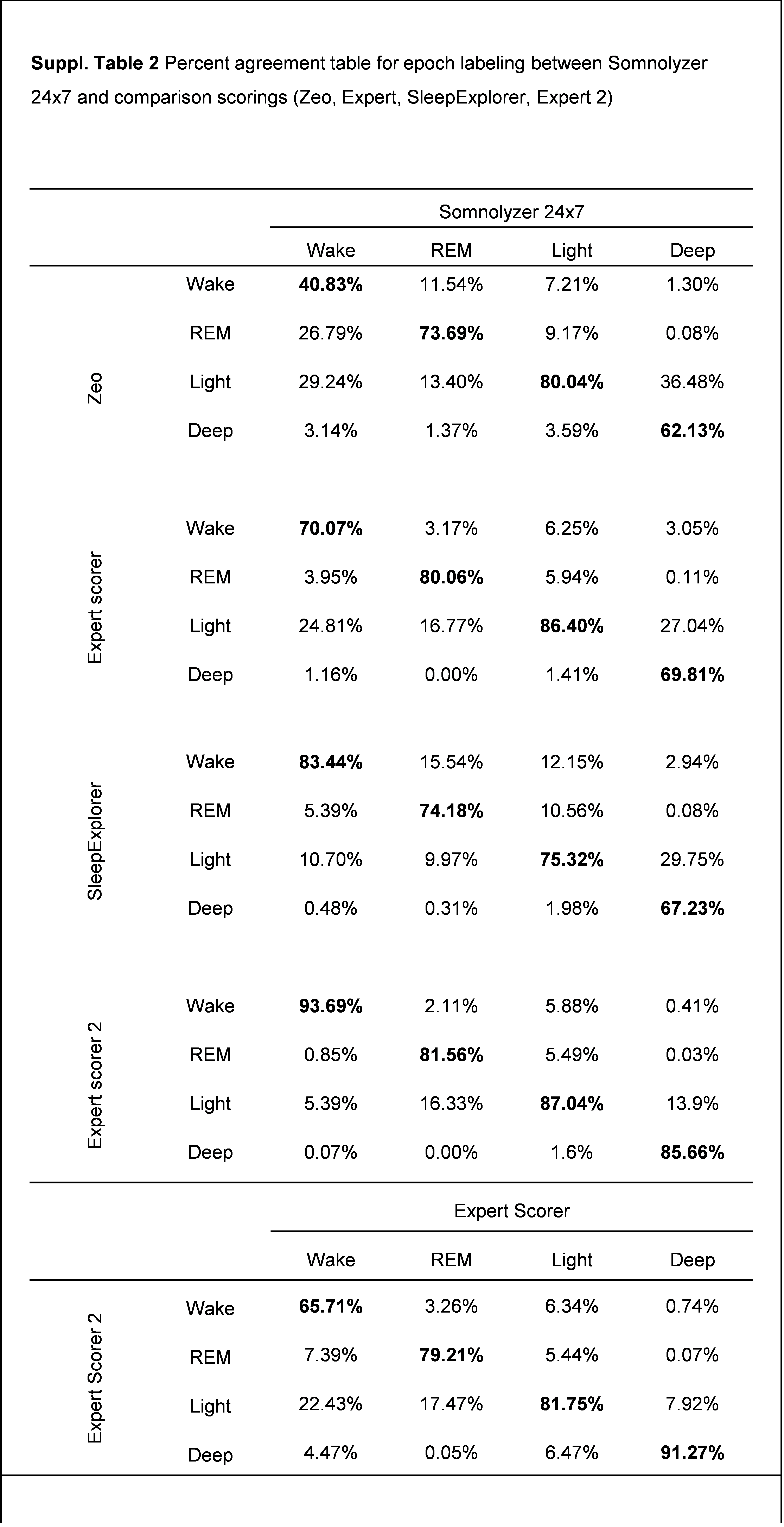

Supplement: Supplementary file 6 — High resolution image (TIFF 2280 kb) [file 11325_2012_757_MOESM3_ESM.tif]
